# A Fatal Brain Stroke in Patient with Advanced Breast Cancer Treated with Bevacizumab: A Case Report

**DOI:** 10.4021/wjon265w

**Published:** 2011-01-01

**Authors:** Germano Domenico, Tinessa Vincenza, Barletta Emiddio, Turitto Dino, Muccio Carmine Franco, Daniele Bruno

**Affiliations:** a Oncology Unit, “A.O. G. Rummo”, Benevento, Italy; b Neuroradiology Unit, “A.O.G. Rummo”, Benevento, Italy

**Keywords:** Bevacizumab, Hypertension, Brain stroke

## Abstract

Bevacizumab is a recombinant monoclonal antibody against vascular endothelial growth factor (VEGF) that is used to treat metastatic cancers of the colon, rectum, kidney, and breast. Its side effects include proteinuria, hypertension, gastrointestinal perforations, and arterial emboli. Although these toxic effects are more frequent in patients with atherosclerosis, their pathophysiology remains unresolved. We observed that patients treated with bevacizumab who developed hypertension had similar clinical presentations and biologic features, leading us to propose a unique mechanism for the vascular side effects of bevacizumab. We report a case of a woman treated for her metastatic breast cancer with first line chemotherapy with Paclitaxel plus bevacizumab who developed a brain stroke.

## Introduction

Bevacizumab (Avastin, Genentech, Inc, South San Francisco, CA) is a recombinant monoclonal antibody against vascular endothelial growth factor (VEGF) that is used to treat metastatic cancers of the colon, rectum, kidney, and breast. Its side effects include proteinuria (35% of patients), hypertension (15% – 30% of patients), gastrointestinal perforations (5% – 7% of patients), and arterial emboli (0.5% – 1% of patients) [1–3]. Although these toxic effects are more frequent in patients with atherosclerosis, their pathophysiology remains unresolved. We observed that patients treated with bevacizumab who developed hypertension had similar clinical presentations and biologic features, leading us to propose a unique mechanism for the vascular side effects of bevacizumab.

We report a case of a woman treated for her metastatic breast cancer with first line chemotherapy with Paclitaxel plus bevacizumab who developed a brain stroke.

## Case Report

A 55-year-old woman underwent quadrantectomy for ductal carcinoma of the left breast (pT1pN2cM0 HER2 -, ER +, PgR +) in 1997. After she had adjuvant chemotherapy with FEC for 6 months, then started Tamoxifen for 5 years until 2003.

Routine follow-up was negative until December 2008 when, for bone pain, a bone scan showed lesion in the skull and lombar vertebras. A CT scan showed small lesions in the liver.

For this reason the patient started chemotherapy with Paclitaxel (80 mg/m^2^ weekly) plus Bevacizumab (10 mg/kg every 2 weeks). Chemotherapy was delivered for 6 months with good compliance (Alopecia G3, Neutropenia G2), therefore, restaging of disease showed a stable disease, so the patient took maintenance therapy with Bevacizumab every 2 weeks.

After the 4th infusion of maintenance therapy, the patient was admitted to Emergency Room of our hospital for a stroke.

**Figure 1:**
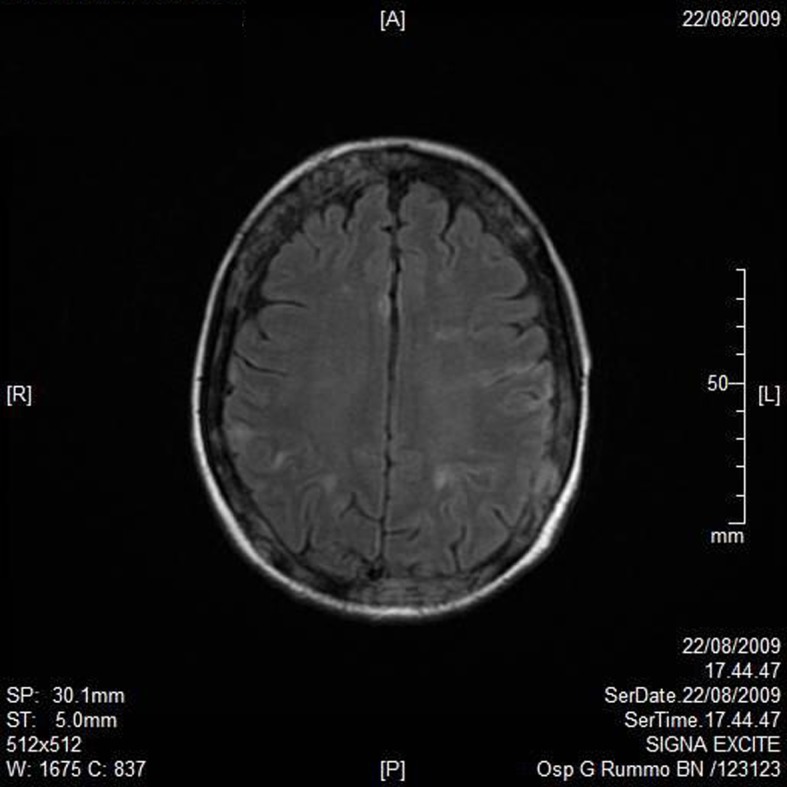
Axial T1 imaging.

**Figure 2:**
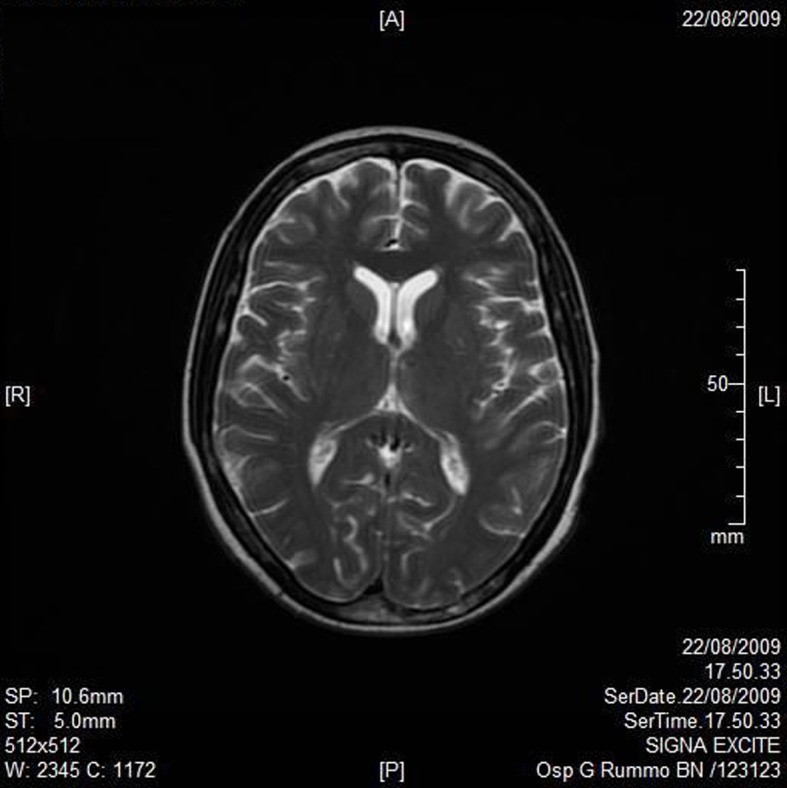
Axial T2W imaging.

A RMN of the brain showed this clinical imaging (Fig. 1, 2) as a multinfartual area due to microembolization in blood vessels of the brain. The patient died after 10 days.

## Discussion

VEGF is a well-known antihypertensive agent [4]. Thus, inhibition of the VEGF pathway with bevacizumab could in theory lead to hypertension. However, in patients treated with sorafenib, another anti-VEGF agent, there was no correlation between the development of hypertension and serum VEGF level [5]. Moreover, serum catecholamine, rennin, and aldosterone levels did not change during anti-VEGF therapy, lessening the likelihood that the onset of hypertension had an adrenergic or a renovascular etiology.

We hypothesize that bevacizumab induced VEGF inhibition could be responsible for CES. In the subset of patients with atherosclerosis, CES may account for all bevacizumab-induced acute complications, including hypertension, arterial emboli, gastrointestinal perforations (related to mesenteric ischemia), and late-onset renal dysfunction [5].

In view of this observation, a prospective follow-up of atherosclerotic patients receiving bevacizumab seems warranted.

Breast cancer represents a heterogeneous array of different disease subtypes with unique molecular phenotypes and distinct clinical feature. Despite advances in the treatment of early-stage breast cancer, approximately one third of patients will eventually develop metastatic breast cancer (MBC) (Early Breast Cancer Trialists’ Collaborative Group 1998). The prognosis of patients with MBC is poor, with a median survival time ranging from 24 to 48 months. Recently, advances in understanding the biology of breast cancer have led to the classification of breast tumors based upon their molecular features and the advent of targeted therapies for the treatment of both early and MBC. Targeted agents and their promise of better patient outcome with respect to safety, survival, and quality of life may change the clinical course for many MBC patients. VEGF is a critical mediator in tumor angiogenesis for many solid malignancies, including breast cancer. Upon binding to its receptor, VEGF induces a cascade of intracellular signals inducing cellular proliferation, increased vaso-permeability, inhibition of apoptosis, and ultimately angiogenesis. For many tumors, VEGF appears to be a rate-limiting signal in angiogenesis, making it an attractive target for therapeutic agents. Bevacizumab is a humanized recombinant antibody that prevents VEGF receptor binding, and inhibits angiogenesis and tumor growth. Its use has been recently approved in the US and in the EU for colorectal cancer, breast cancer, non-small cell lung cancer, and renal cell carcinoma (Genentech Inc., European Medicines Agency). Bevacizumab appeared safe and manageable in patients with MBC, with minimal additional toxicity seen when combined with other agents. However, the addition of bevacizumab increased the incidence of hypertension and proteinuria. Additional severe adverse events have been reported with bevacizumab: hypertension, including hypertensive crisis; proteinuria, including nephritic syndrome; thrombosis (venous and arterial), including cerebral and myocardial infarction, transient ischemic attacks, and deep venous thrombosis; bleeding and hemorrage; impaired wound healing; gastrointestinal perforation; congestive heart failure (seen only in patients receiving anthracyclines and/or left chest-wall irradiation). These events are exceedingly rare, but do warrant consideration in selected patients for whom bevacizumab is planned. Venous and arterial thrombotic events also have been reported in other trials, including cerebral and myocardial infarction and pulmonary embolism. Clinicians should consider avoiding bevacizumab in patients with a history of prior thrombotic events and in patients receiving systemic anticoagulation, because the combination has not been studied in MBC.

The interactions between cancer and thrombosis are multiple and well documented. The cancer population has an overall 4.3-fold increased risk of thrombosis in the Multiple Environmental and Genetic Assessment study [6], and patients with idiopathic venous thrombosis have an increased risk of harboring an occult malignancy. The procoagulant activity of cancer cells is often associated with the expression of tissue factor (TF) and subsequent activation of protease receptors on cell surfaces followed by fibrin generation and the release of vascular growth factors. TF has been described in a wide variety of tumor cells, and its expression correlates with disease stages and survival. Thrombotic events were reported in up to 23% of patients using bevacizumab in combination with chemotherapy for colorectal and gastric cancers, whereas up to 16% of patients treated with bevacizumab in combination with carboplatin and paclitaxel for non-small-cell lung cancer experienced grade 3 and 4 bleeding episodes. Discrepancies in toxicities observed in similar population studies make it difficult to define the causal role of bevacizumab in the development of vascular events. For instance, two randomized trials in metastatic colorectal cancer comparing similar chemotherapy regimens with or without the addition of bevacizumab have shown a different toxicity profile of the experimental arm. Hurwitz et al [1] observed only an insignificant increase in thrombotic or bleeding events, whereas, in the trial by Kabbinavar et al [7], thrombosis was the most frequent toxicity; in particular, in the lowdose arm, bleeding episodes, even if mild (mostly grade 1 or 2), were observed in 53% of patients. Also, a significant difference in thrombotic rate between the low- and high-dose bevacizumab arms cannot be easily explained. These apparently disparate toxicities (thrombosis and bleeding) can both theoretically result from endothelial cell perturbations induced by the drug, such as nonphysiologic endothelial cell apoptosis as a result of inhibition of VEGF. This hypothesis is based on the observation that VEGF also has a maintenance role for the normal endothelium function [8]. Abnormal apoptosis of endothelial cells can lead to exposure of the highly prothrombotic basement membrane and, at the same time, cause loss of integrity of the endothelial vessel lining with subsequent hemorrhage. The prothrombotic effect of anti-VEGF agents may also derive from a platelet-dependent mechanism. In fact, VEGF signaling seems essential for the production of the platelet inhibitors prostaglandin I-2 and nitric oxide. Reduced levels of prostaglandin I-2 and nitric oxide may lead to increased platelet activation and increased incidence of ATE.
